# A complete posterior tibial stress fracture that occurred during a middle-distance running race: a case report

**DOI:** 10.1007/s00402-018-3035-5

**Published:** 2018-09-07

**Authors:** Jun Komatsu, Atsuhiko Mogami, Hideaki Iwase, Osamu Obayashi, Kazuo Kaneko

**Affiliations:** 1 Departments of Medicine for Motor Organs, Juntendo University Graduate School of Medicine, 2-1-1 Hongo, Bunkyo-ku, Tokyo, 113-8421 Japan; 2 Department of Orthopaedic Surgery, Juntendo University Shizuoka Hospital, 1129 Nagaoka, Izunokuni, 410-2295 Shizuoka Japan; 3 Department of Bio-Engineering, Juntendo University Institute of Casualty Center, 1129 Nagaoka, Izunokuni, 410-2295 Shizuoka Japan

**Keywords:** Stress fractures, Running, Overuse injuries, Posterior tibial stress fracture, Runner-type stress fracture, Intramedullary nailing

## Abstract

Posterior tibial stress fractures are more frequent than anterior tibial stress fractures, and they are considered to have a good prognosis for returning to sports; cases leading to a complete fracture are rare. A 17-year-old male involved in high school athletics middle-distance running had a 3-week history of pain with training. He was running up to 300 km/week on streets and cross-country in an even distribution. He had posterior tibial stress fractures, but despite the lower leg pain, he continued running. One year later, he was brought to the emergency department after having sustained an injury to the right lower leg while running in a middle-distance race; bilateral tibial stress fractures, with one side complete and the opposite side incomplete, had developed simultaneously. This relatively rare case of bilateral posterior stress fractures, with one side a complete fracture and the opposite side an incomplete fracture, that was treated surgically via exchange intramedullary nailing is reported. The patient could begin light jogging from 3 months after surgery and was without symptoms at 5 months after surgery. He could resume middle-distance racing after 1 year. Posterior tibial cortical fractures are more common and respond better to conservative treatment than anterior tibial stress fractures, and they are a common fracture type in runners. We believe that close, careful follow-up is necessary if patients continue excessive training.

## Introduction

Highly committed athletes commonly develop stress fractures. In the general population, long-distance running is a particularly common form of exercise, physical activity, and leisure activity. It has become increasingly popular due to its easy accessibility and a growing interest in disease prevention. Although many positive health effects have been attributed to distance running, it can cause injuries. Repetitive tissue stress frequently causes overuse injuries affecting the lower extremities [1]. Adolescent athletes place high physical demands on their bodies that vary depending on the given sports activity, which may cause stress or avulsion fractures due to repetitive microtrauma that overloads the bone. A runner who suddenly increases the intensity and duration of training is at risk for developing a stress fracture [2]. A tibial shaft stress fracture is the most frequent such fracture in athletes [3, 4]. The tibia is reported to be the most common site of stress fractures, accounting for 35–56% of all stress fracture injuries [5].

Tibial stress fractures can be classified into two groups depending on the location, anterior and posterior, causing anterior and posterior/posteromedial stress fractures, respectively. Anterior stress fractures occur in sports with frequent jumping, and they are characterized by prolonged healing due to excessive fibrous growth [6]. Anterior cortical fractures are less common than posterior stress fractures [7], and they often heal poorly due to constant tension exerted by relatively poor vascular and posterior muscular forces; they are located on the anterior, tension side of the tibial shaft, and are prone to delayed union and nonunion [8]. In some instances, anterior tibial stress fractures can progress to complete fractures [9–11].

On the other hand, posterior tibial cortical fractures are more common and respond adequately to conservative treatment, but they are a significant clinical problem for runners. In most cases, conservative treatment is sufficient, and surgical treatment is very rarely needed. The most predominant type is the low-risk posteromedial cortex (compression side) stress fracture [3, 12, 13]. However, to the best of our knowledge, no previous reports have specifically examined complete posterior tibial stress fractures.

A relatively rare case of simultaneous bilateral posterior tibial stress fractures, in which one side was a complete fracture and the opposite side was an incomplete fracture, which was treated surgically via exchange intramedullary nailing, is reported.

## Case report

**Fig. 1:**
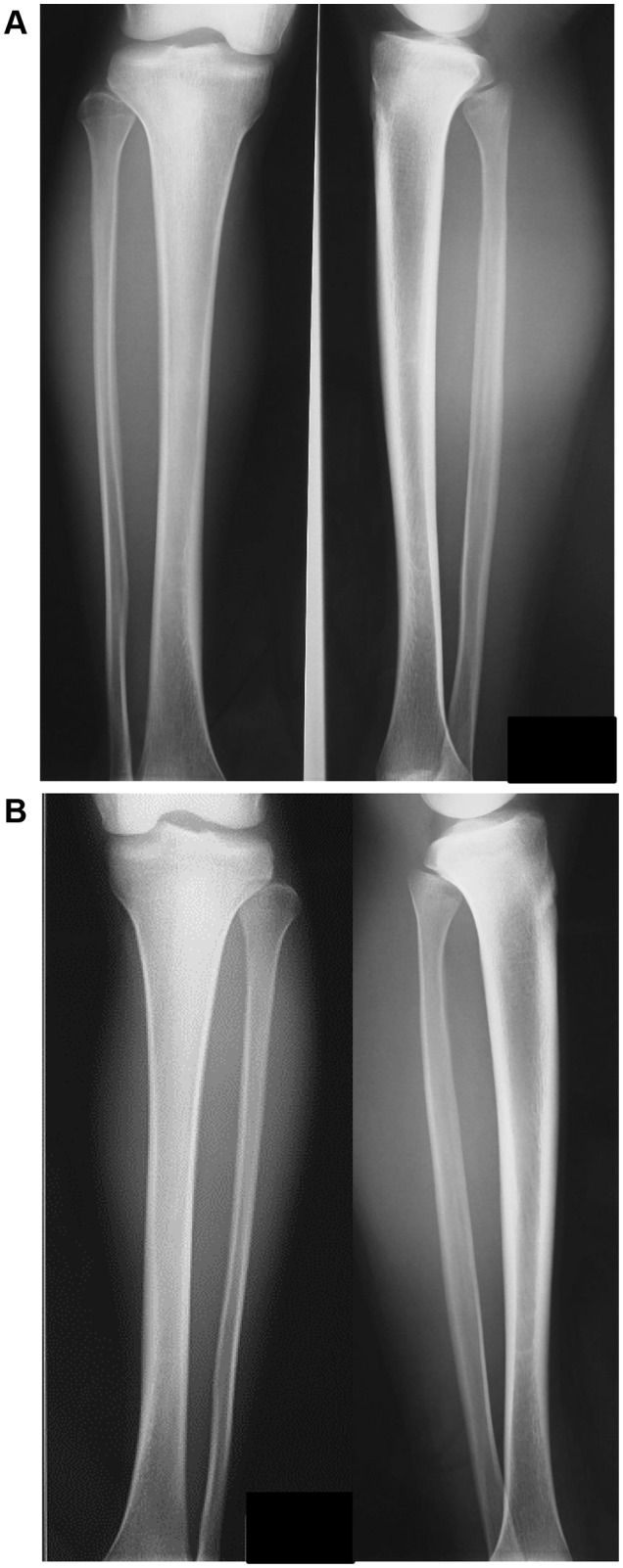
Initial radiographs show suspected tibial stress fracture or shin splints. There is no clear abnormality in the radiographs of the tibia in the antero-posterior and lateral views. **A** Right side; **B** left side

**Fig. 2:**
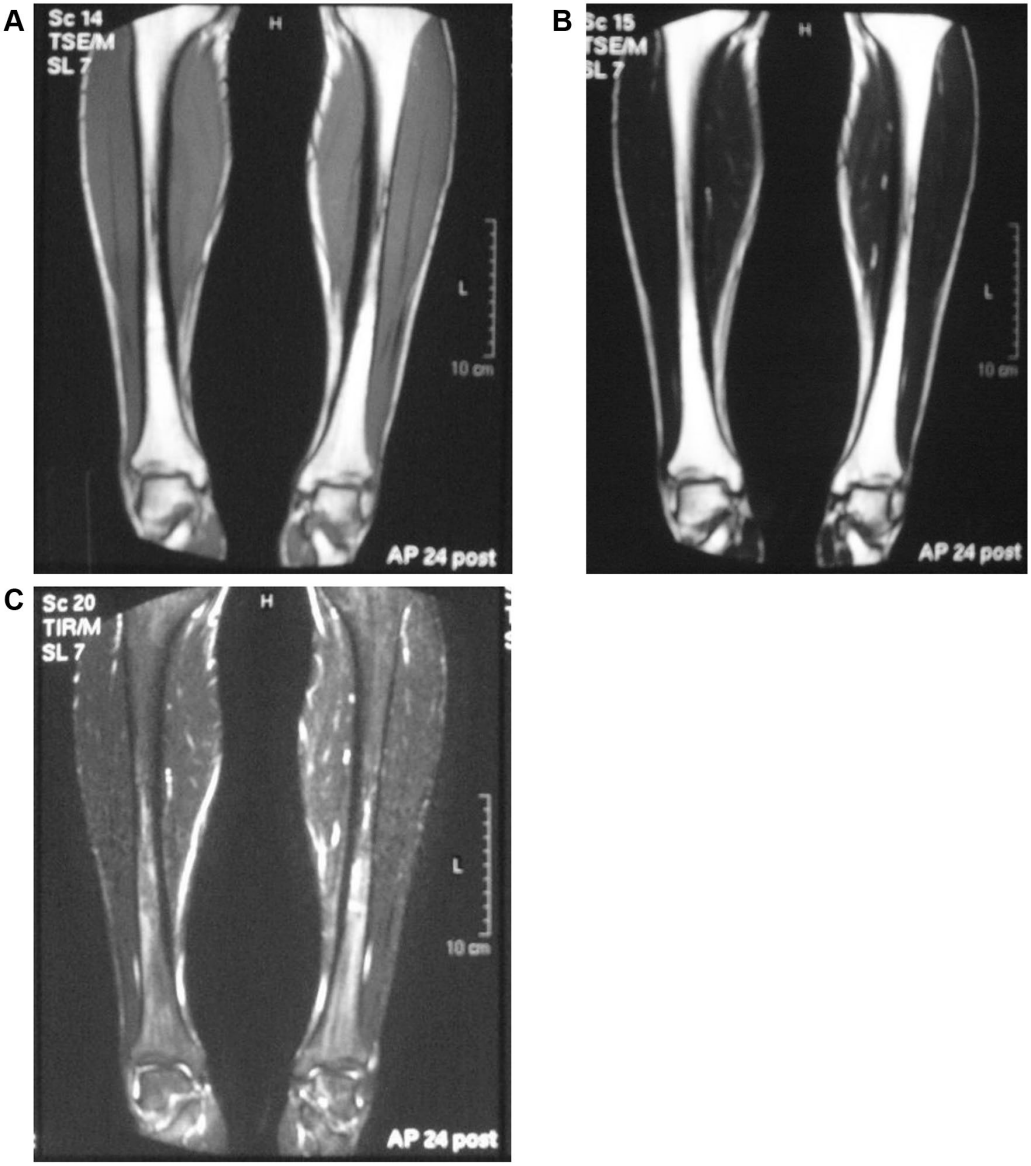
Initial coronal MRI scans diagnosed with a stress fracture show a strikingly wide low-signal intensity on the T1-weighted scan (**A**), and a high-signal intensity on the T2-weighted scan (**B**) and STIR fat-suppressed scan (**C**) in the localized bone marrow. The abnormal finding is more detectable on the STIR fat-suppressed MRI scan

**Fig. 3:**
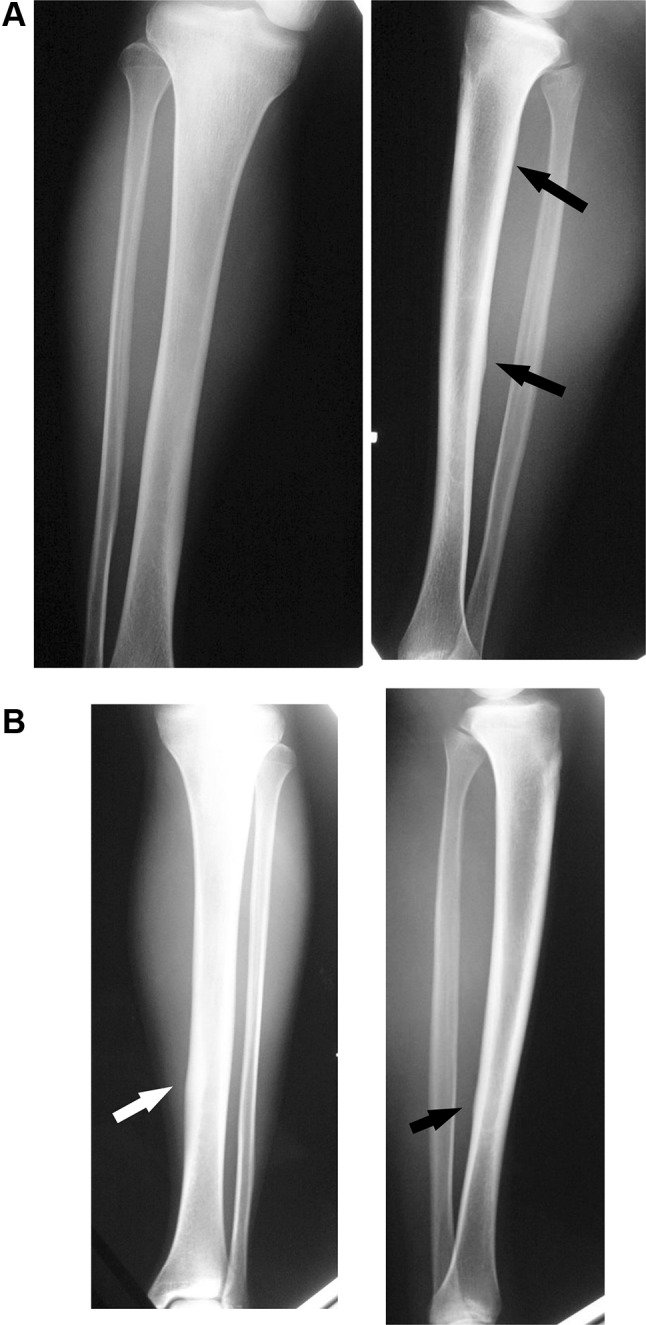
Radiographs of both legs 6 months after the initial visit to our hospital. Obtained 6 months after the first examination, callus formation is seen at the lateral and posterior side of the tibia in the antero-posterior and lateral views. The arrows indicate callus formation at the lateral and posterior sides of the tibia

A 17-year-old male involved in high school athletics middle-distance running presented with a 3-week history of pain with more training. He was running up to 300 km/week on streets and cross-country in an even distribution. Although he had taken analgesics, the pain during exercise did not improve, and he presented to our emergency department with lower leg pain (Fig. 1). There was no clear abnormality on the radiographs of the tibia, but STIR magnetic resonance imaging (MRI) confirmed a high-intensity area of the distal one-third of the tibia, and the diagnosis of stress fracture and shin splint was made. The patient was instructed to suspend training, and the injury was treated conservatively with follow-up on an outpatient basis (Fig. 2). Follow-up radiographs were checked at 2 and 3 months. With this treatment, the fracture healed with no complications, and he decided to return to running after 3 months. At 6 months, radiography showed thickening of the bone cortex in the back one-third of the right tibia and in the back of the distal part of the left tibia, so that he was again instructed to stop training (Fig. 3). However, he discontinued coming to the outpatient clinic on his own after 6 months.

**Fig. 4:**
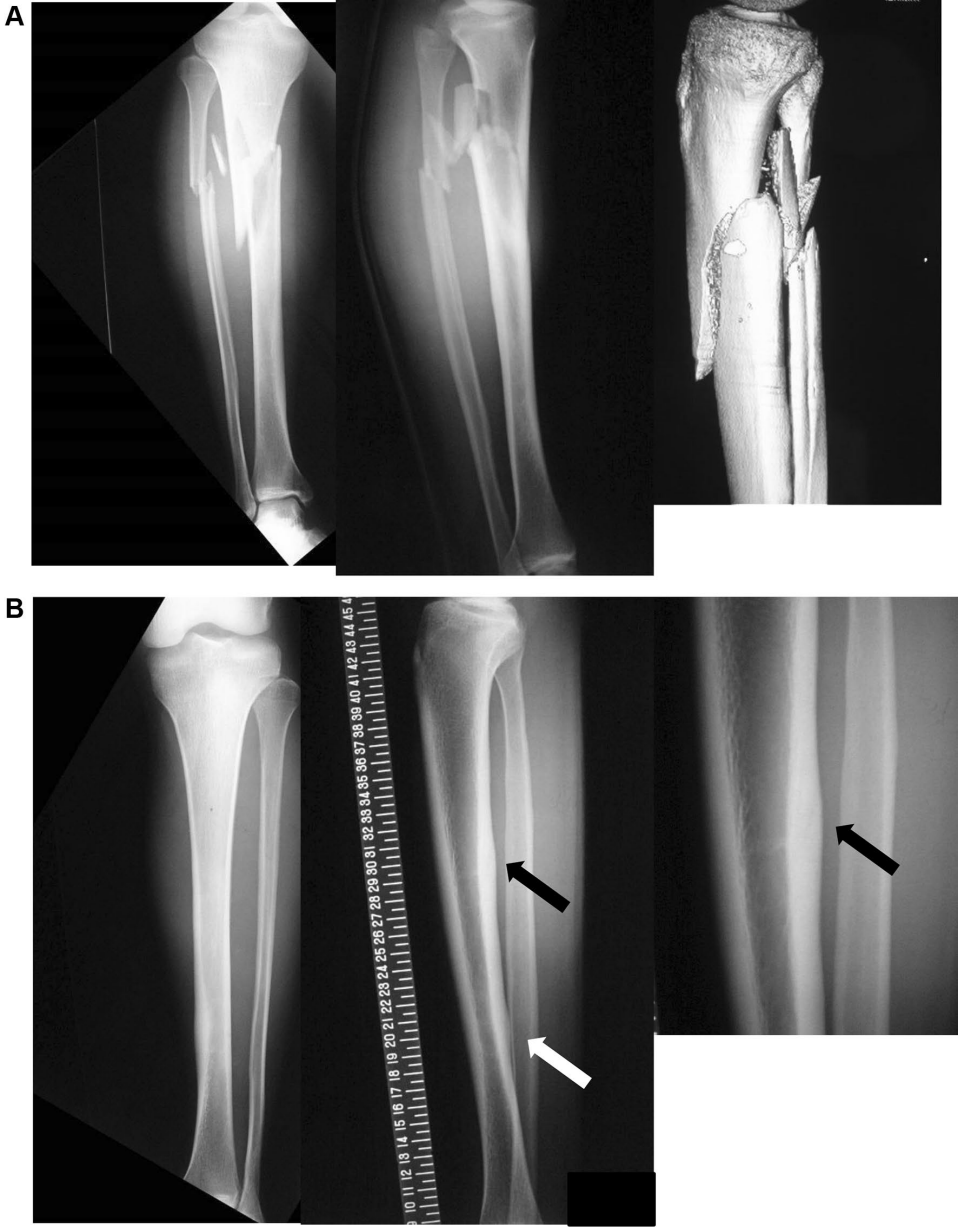
Radiographs at the emergency department with deformity of the right lower leg. Full-length tibial radiographs were requested in keeping with the clinical picture, and they confirm complete tibial and fibular fractures of the right side. **A** Right-side tibial radiography and 3D computed tomography; **B** Left-side tibial posterior stress fracture. Arrows indicate callus formation sites

He was then seen in the emergency department, having sustained an injury to the right lower leg while running a middle-distance race, 1 year after the initial examination. He described how, when he had just started and passed through the first corner, he had felt a ‘‘snap’’ in his right calf, suddenly could not run, and fell and had to abandon the race. He said that his leg was deformed in an impossible direction. It became impossible to run because of the lower leg deformities, and he was brought to our emergency department. He was admitted to hospital, and X-ray examination showed a greatly displaced oblique fracture in the proximal 1/3 of the left lower leg, but, fortunately, there was no open wound (Fig. 4). Before the race that day, the patient had not experienced any pain or discomfort in his left lower leg while running. In the emergency department, the primary assessment showed moderate direct and indirect tenderness of the opposite proximal tibia. There was no malalignment, swelling, or discoloration of the left lower leg.

**Fig. 5:**
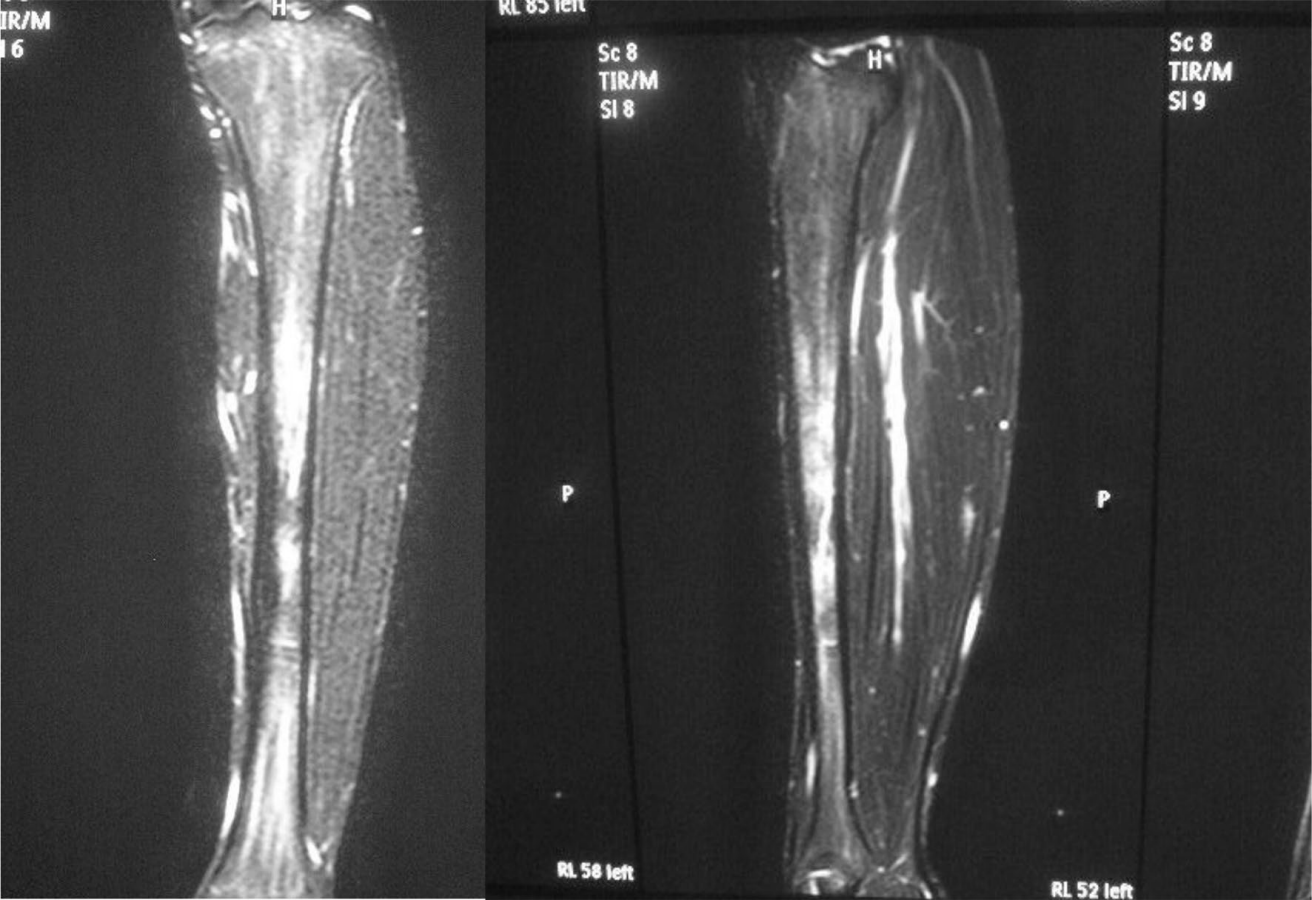
MRI of the left lower leg after right complete fractures. On MRI, STIR shows an abnormal high signal

**Fig. 6:**
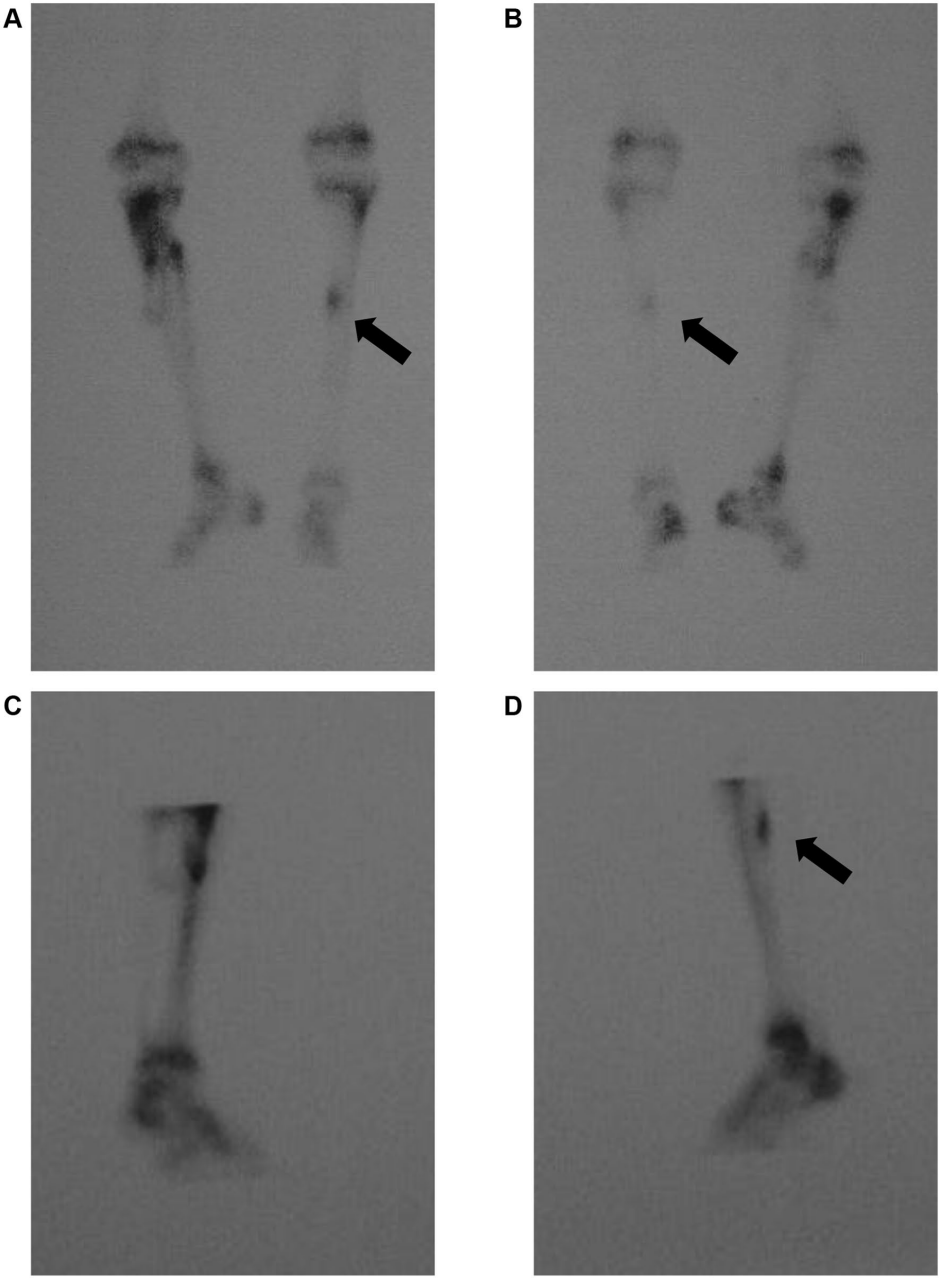
Bone scintigraphs show abnormal local uptake in the antero-posterior (**A**) and postero-anterior (**B**) and lateral views of the right side (**C**) and left side (**D**) of the patient with stress fractures. Arrows indicate longitudinal linear uptake in the bone scintigraph views of the patient with stress fractures

The radiographs showed a full-thickness fracture of the proximal one-third posterior tibial shaft (Fig. 4). On MRI, T1-weighted imaging showed a high-signal area at the middle one-third and a low-signal on T2-weighted imaging, and STIR showed an abnormal high signal at the same site (Fig. 5). These findings suggested abnormalities such as edema and bleeding in the bone marrow. On bone scintigraphy, there was moderate accumulation in the vicinity of the left lower third of the thigh, so a left tibial stress fracture was diagnosed (Fig. 6).

**Fig. 7:**
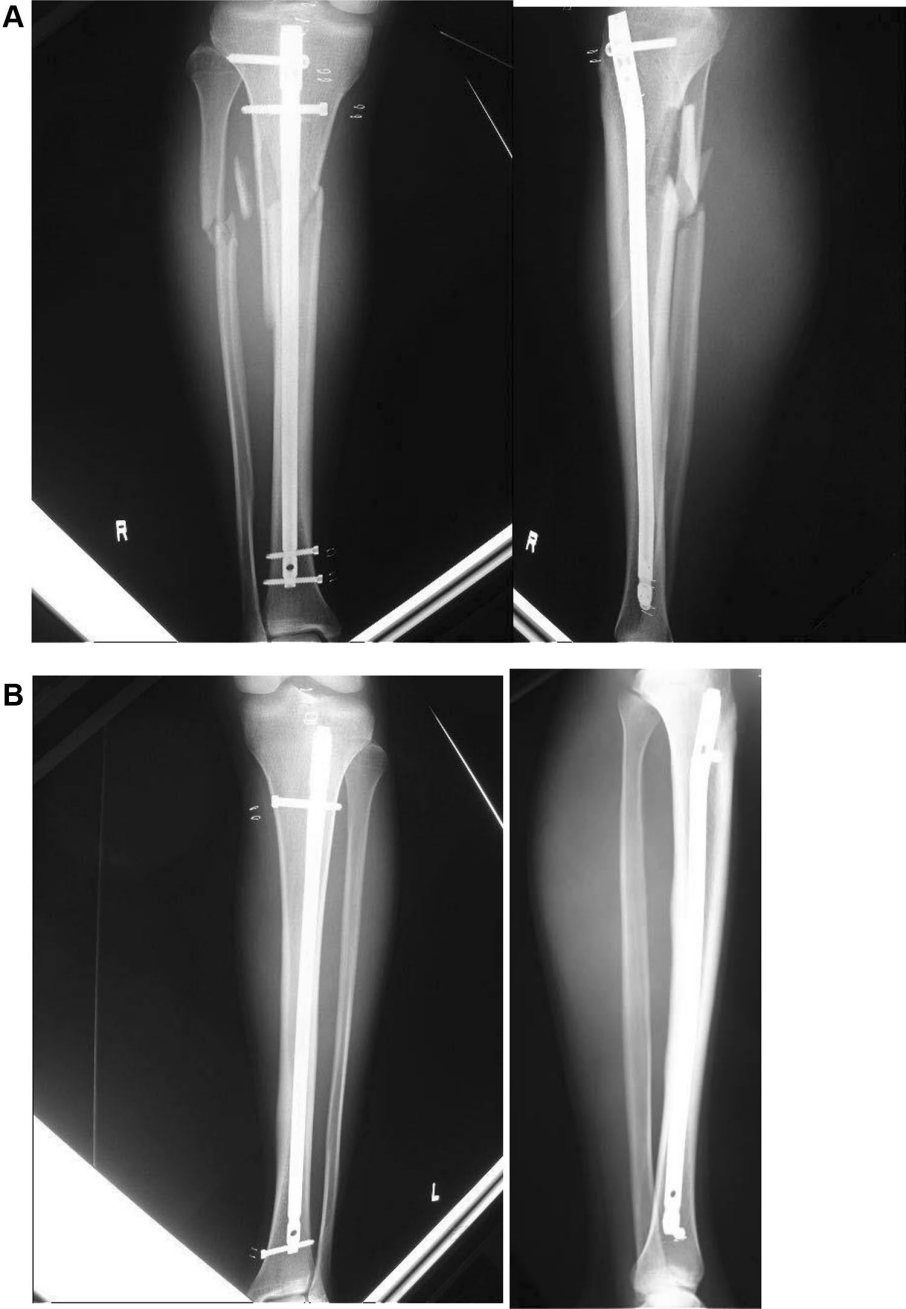
Radiographs show fixation of both tibial fractures, complete and incomplete, with intramedullary nailing. Closed reduction and internal fixation of the radial shaft fracture were performed using intramedullary nailing for both the right complete tibial fracture (**A**) and the left tibial stress fracture (**B**)

The following day, surgical treatment for the injury was performed under general anesthesia after written, informed consent was obtained from the patient and parents. In addition, the patient gave written, informed consent for publication of the case, including the accompanying images. At our institution, ethical approval is not required for reporting individual cases. Closed reduction and internal fixation of the radial shaft fracture were performed, with intramedullary nailing (10-mm-diameter, T2 Nailing system, Stryker, Kalamazoo, MI) for both the left complete tibial fracture and the right tibial stress fracture (Fig. 7).

**Fig. 8:**
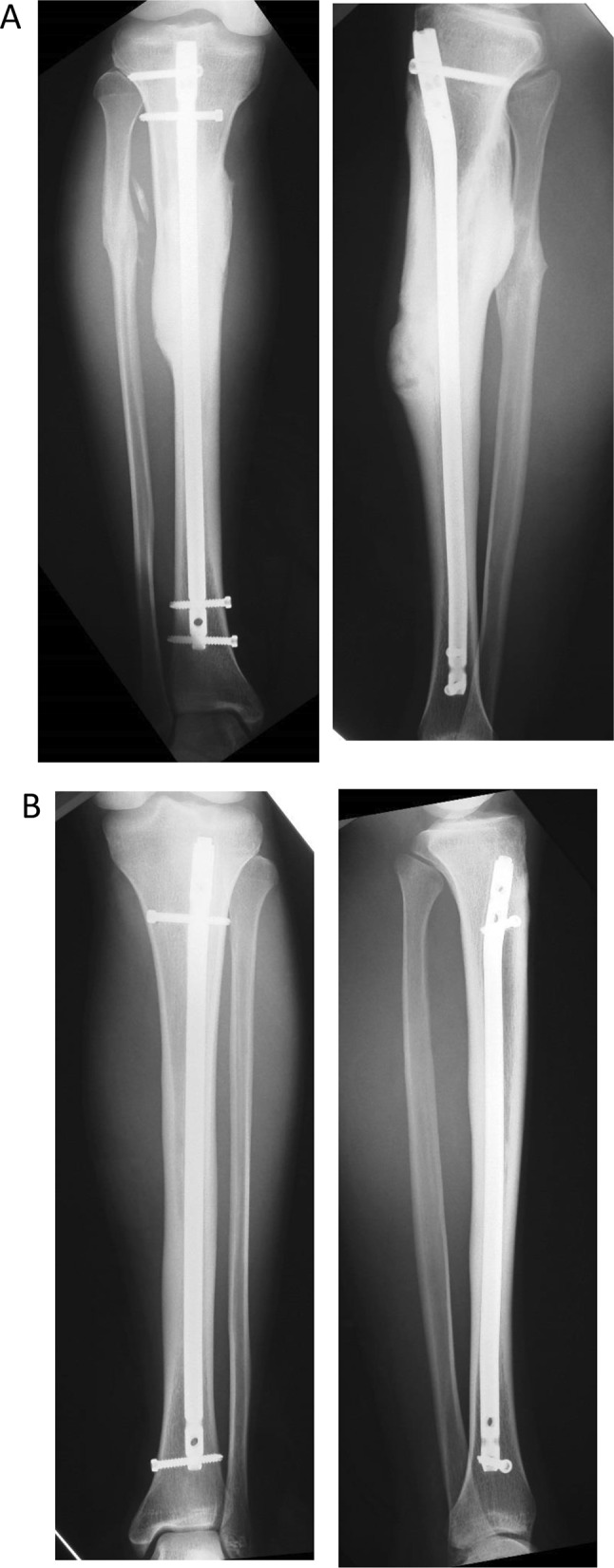
Postoperative antero-posterior and lateral radiographs show newly healed tibial stress fractures. Postoperative radiographs show antero-posterior and lateral low views. The patient was asymptomatic and clinical healing of the fracture is apparent 10 months after the nailing, and a fracture line is hardly visible on the radiographs. **A** Right side; **B** left side

The injury was then treated conservatively, with 9 weeks in an ROM knee cast and no weight-bearing on the affected leg. Healing was monitored through a series of follow-up radiographs. With this treatment, the fracture healed with no complications. Although the patient was asymptomatic and clinical healing of the fracture was apparent 10 months after the nailing, a fracture line was still visible on radiographs (Fig. 8). The patient could begin light jogging from 3 months after the operation and was without symptoms at 5 months. He returned to middle-distance racing after 1 year.

## Discussion

If operative treatment is not performed, a stress fracture that is persistently painful may develop delayed union, nonunion, or even a complete fracture. An established technique for treating delayed or nonunion tibial stress fractures is intramedullary nailing [14]. There are two types of tibial stress fractures, anterior and posterior, based on their location. Furthermore, there are many frequent running-type stress fractures (posterior fractures), which are thought to resolve better than jumping-type stress fractures (anterior fractures), which often develop in field athletes. In particular, posterior tibial cortical fractures, which are more frequent and show an adequate response to conservative therapy, are often seen in runners. Ohnishi said that actually they were widely distributed proximally to distally including the middle third, so runner-type stress fractures are more likely to be generated from the posterior tibia [15]. The distance run per week can also be a factor in stress injuries. It has been shown that running more than 64 km/week (approximately 40 miles/week) is a significant risk factor for lower extremity injuries [16].

In the present case, the patient had posterior tibial fractures, but, despite the lower leg pain, he continued running, so that bilateral tibial stress fractures, with one side complete and the opposite side incomplete, occurred simultaneously. To the best of our knowledge, there have been no previous reports of a posterior tibial stress fracture with a complete fracture as in the present case. Because the symptoms improve with rest for a short period of time, many patients may not seek treatment. Moreover, there may be intrinsic elements, that are internal factors, which result in additional stresses to the bone. Such intrinsic elements include anatomical variations, footwear, running mechanics, training regimens, and running surfaces, as well as individual health factors, such as poor bone health (osteoporosis and low bone density) [17]. According to Reeder et al. [18], it is important to focus on the runner’s training regimen and history to identify potential injury-causing factors. A pes cavus foot is linked to stress fracture incidence; because this foot type is more rigid, it does not absorb shock and passes impact forces to the tibia, therefore, increasing the risk for a tibial stress injury [19].

Most patients with the symptoms of stress fractures reduce the distance, frequency, and intensity of their activities. Distance runners have an increased risk for stress fractures because of the high impact and repetitive loads. With repetitive mechanical loading of bone, cumulative bone strain can cause bone damage and stress fractures if net bone damage is chronically greater than bone repair [20]. Especially in runners, posterior tibial stress fractures occur commonly on the compressive posterior surface of the proximal and distal thirds of the tibia; these fractures can be considered low-risk fractures compared to anterior fractures and managed by relative rest. Nonsurgical treatment of posterior tibial stress fractures begins with rest and stopping the aggravating activity. A stress fracture is a mechanical failure of the bone in which the activity of the osteoblasts cannot keep pace with the activity of the osteoclasts. Repetitive, cyclical loading of the bone with inadequate recovery occurs, and the bone is unable to repair itself between exercise sessions [18]. With heavy loading of a bone, microcracks may appear within the bone tissue. Such microcracks are thought to contribute to activating the remodeling process, which is required for adaptation of the bone to the functional demands on the tissues caused by loading. However, with excessive loading, either in magnitude or frequency, a stress fracture can develop due to the insufficient time for remodeling to repair the microcracks [6]. Unfortunately, the repetitive and high loading nature of running creates an ideal environment for the development of stress fractures. Furthermore, a complete fracture may occur due to large cracks.

In the present case, due to repeated periods of rest and resumption of competition, at 1 year, a complete fracture finally resulted. Restricting excessive exercise and ensuring sufficient rest when lower extremity pain appears may have made it possible to return to racing at an early stage. The present case had an insidious onset, presenting with moderate clinical signs and symptoms; therefore, diagnosis might have been delayed by analgesic medication, resulting in a complete fracture. At the same time, intramedullary nailing was performed for the opposite tibial stress fracture to facilitate an early return to training.

In conclusion, our observations demonstrate that the posterior tibia stress fracture is a more frequent tibial stress fracture than the anterior stress fracture, and it is considered to have a good prognosis for returning to sports; although rare, there are cases that result in a complete fracture, and when excessive training is continued, careful follow-up is needed.
